# Acetate supplementation modulates brain histone acetylation and decreases interleukin-1β expression in a rat model of neuroinflammation

**DOI:** 10.1186/1742-2094-9-51

**Published:** 2012-03-13

**Authors:** Mahmoud L Soliman, Mark D Smith, Heidi M Houdek, Thad A Rosenberger

**Affiliations:** 1 Department of Pharmacology, Physiology and Therapeutics, University of North Dakota School of Medicine and Health Sciences, Grand Forks, North Dakota 58203, USA

**Keywords:** Acetylation, brain, cytokines, histone, histone acetyltransferase, histone deacetylase, neuroinflammation

## Abstract

**Background:**

Long-term acetate supplementation reduces neuroglial activation and cholinergic cell loss in a rat model of lipopolysaccharide-induced neuroinflammation. Additionally, a single dose of glyceryl triacetate, used to induce acetate supplementation, increases histone H3 and H4 acetylation and inhibits histone deacetylase activity and histone deacetylase-2 expression in normal rat brain. Here, we propose that the therapeutic effect of acetate in reducing neuroglial activation is due to a reversal of lipopolysaccharide-induced changes in histone acetylation and pro-inflammatory cytokine expression.

**Methods:**

In this study, we examined the effect of a 28-day-dosing regimen of glyceryl triacetate, to induce acetate supplementation, on brain histone acetylation and interleukin-1β expression in a rat model of lipopolysaccharide-induced neuroinflammation. The effect was analyzed using Western blot analysis, quantitative real-time polymerase chain reaction and enzymic histone deacetylase and histone acetyltransferase assays. Statistical analysis was performed using one-way analysis of variance, parametric or nonparametric when appropriate, followed by Tukey's or Dunn's post-hoc test, respectively.

**Results:**

We found that long-term acetate supplementation increased the proportion of brain histone H3 acetylated at lysine 9 (H3K9), histone H4 acetylated at lysine 8 and histone H4 acetylated at lysine 16. However, unlike a single dose of glyceryl triacetate, long-term treatment increased histone acetyltransferase activity and had no effect on histone deacetylase activity, with variable effects on brain histone deacetylase class I and II expression. In agreement with this hypothesis, neuroinflammation reduced the proportion of brain H3K9 acetylation by 50%, which was effectively reversed with acetate supplementation. Further, in rats subjected to lipopolysaccharide-induced neuroinflammation, the pro-inflammatory cytokine interleukin-1β protein and mRNA levels were increased by 1.3- and 10-fold, respectively, and acetate supplementation reduced this expression to control levels.

**Conclusion:**

Based on these results, we conclude that dietary acetate supplementation attenuates neuroglial activation by effectively reducing pro-inflammatory cytokine expression by a mechanism that may involve a distinct site-specific pattern of histone acetylation and histone deacetylase expression in the brain.

## Background

Reversible epigenetic changes play a major role in regulating gene expression in the post-mitotic brain. The most prominent mechanism involved in this process is the alteration in histone acetylation, which is known to influence development, differentiation and the injury response [1]. Therefore, understanding the functional consequences of changes in histone acetylation in the brain is important given the impact that it can have on neuroinflammation. Histone proteins are instrumental in the packaging of DNA and play a central role in transcription through a process of acetylation that regulates the accessibility of DNA to proteins involved in transcription. As a general consensus, an increase in histone acetylation is associated with active gene expression while a decrease in histone acetylation is associated with gene repression [2,3]. In this regard, site-specific acetylation patterns have been linked to both physiological and pathological roles. For example, histone H4 acetylated at lysine 16 (H4K16) is essential for transcription initiation [4] and DNA repair [5]. Histone H3 acetylated at lysine 9 (H3K9) is selectively enriched at the promoters of stem cells, suggesting a role in pluripotency [6].

The histone acetylation state is actively maintained by the opposing activities of two enzyme families: histone acetyltransferases (HATs) and histone deacetylases (HDACs). Based on structural homology, HDACs are classified into different classes: HDAC class I are mainly located in the nucleus, HDAC class II shuttle between the nucleus and cytoplasm [7] and HDAC class III (sirtuins) are located in the cytoplasm. HDAC classes I and II are inhibited by conventional HDAC inhibitors while class III HDACs are nicotinamide adenine dinucleotide (NAD^+^)-dependent and inhibited by nicotinamide [8]. Likewise, HAT are classified into distinct families (general control non-derepressible 5 (GCN5), P300/cyclic adenosine monophosphate response element binding protein associated factor (PCAF), the MYST family named for its founding members in yeast and mammals, monocytic leukemia zinc finger protein (Moz), Something About Silencing protein (Sas2p), and HIV tat-interacting protein 60 (Tip60), transcription initiation factor TFIID 250 kDa subunit (TAFII250), steroid receptor coactivator proteins (SRC), and GCN5-related N-acetyltransferase (GNAT)) that show high sequence similarity within families, but poor-to-no sequence similarity between families [9]. The exact correlation of individual HAT or HDAC with site-specific acetylation or deacetylation of histone lysine residues remains largely unknown due to overlapping enzyme targets [10,11].

Lipopolysaccharide (LPS), an endotoxin present in the membrane of Gram-negative bacilli, binds to toll-like receptor 4 found on brain microglia and promotes an inflammatory response characterized by the enhanced expression of the pro-inflammatory cytokine IL-1β, neuroglial activation and neurodegeneration [12-14]. LPS infusion through a cannula implanted into the fourth ventricle of the brain and connected to a subcutaneous mini-osmotic pump is used as a model to study neuroinflammation in rats [15-17]. In this model, the turnover and metabolism of brain arachidonic acid is increased by 40%, the activities of both arachidonic acid-selective secretory and cytosolic phospholipases A2 increase, as do the levels of prostaglandins E2 and D2 [18,19]. Thus, this model reproduces many of the properties associated with known modalities of neuroinflammation.

We have demonstrated in this model that acetate supplementation, using glyceryl triacetate (GTA), increases brain acetyl-coenzyme A (CoA) levels two-fold and attenuates both neuroglial activation and cholinergic cell loss [17]. Dietary acetate supplementation is suggested as a potential therapy for Canavan's disease [20], is effective in alleviating tremors in a rat model of Canavan's disease [21] and is neuroprotective in a rat model of traumatic brain injury [22]. A single oral dose of GTA given to normal rats differentially increases the proportion of acetylated brain H3K9, H4 acetylated at lysine 8 (H4K8) and H4K16, with no changes in the acetylation state of histone H3 acetylated at lysine 14 (H3K14), histone H4 acetylated at lysine 5 (H4K5) or histone H4 acetylated at lysine 12 (H4K12). It also decreases brain HDAC activity and HDAC2 expression with no changes in brain HAT activity [23].

Since an increase in histone acetylation correlates with anti-inflammatory response and neuroprotection [24,25], and because acetate supplementation alters brain histone acetylation, we propose that long-term acetate supplementation reduces neuroglial activation and cholinergic cell loss by modulating the brain histone acetylation state and pro-inflammatory cytokine expression. This hypothesis is based on the premise that the innate immune response in the brain is dependent on the communication between neuroglia and that an increase in pro-inflammatory cytokine expression will result in heightened microglial and astroglial activation [26]. To begin to test this hypothesis, we quantified the effect that a 28-day-dosing regimen of GTA, used to induce acetate supplementation, had on the brain histone acetylation state, HAT and HDAC enzymic activities, and the expression of the pro-inflammatory cytokine IL-1β. We found that long-term acetate supplementation reversed the LPS-induced decrease in H3K9 acetylation, increased HAT enzymic activity and reduced IL-1β expression in the brain. These results support our hypothesis that long-term acetate supplementation can attenuate LPS-induced changes in histone acetylation and reduce pro-inflammatory gene expression in the brain. The mechanism by which acetate supplementation reduces pro-inflammatory gene expression and neuroglial activation, however, remains to be determined.

## Methods

### Reagents

Antibodies against total histone H3 and H4, and acetylated histone isoforms H3K9, H3K14, H4K5, H4K8, H4K12 and H4K16 were purchased from Upstate Biotechnology (Lake Placid, NY, USA). Antibodies against HDAC1, 2, 3, 4, 5 and 7 were obtained from Cell Signaling Technology Inc. (Danvers, MA, USA). A rabbit polyclonal antibody to IL-1β was purchased from Abcam (Cambridge, MA, USA), and an antibody against α-tubulin and goat anti-mouse immunoglobulin M secondary antibody conjugated with horseradish peroxidase (HRP) were from Santa Cruz Biotechnology, Inc. (Santa Cruz, CA, USA). All Western blot supplies and a goat anti-rabbit HRP-linked antibody were obtained from Bio-Rad Laboratories (Hercules, CA, USA). IL-1β and β-actin primers (mix of reverse and forward primers) were purchased from SA Biosciences (Frederick, MD, USA); FastStart Universal SYBR Green Master was purchased from Roche Applied Science (Indianapolis, IN, USA); an iScript cDNA synthesis kit was purchased from Bio-Rad Laboratories; TRIzol^® ^reagent was purchased from Life Technologies (Grand Island, NY, USA); and nuclease-free water was purchased from Gibco, Life Technologies. Buffering reagents and other chemicals were purchased from EMD Biosciences (Gibbstown, NJ, USA) unless noted otherwise, and GTA was purchased from Sigma (St. Louis, MO, USA).

### Animal surgeries and induction of neuroinflammation

The treatment of all rats used in this study conformed to the Guide for the Care and Use of Laboratory Animals (National Institutes of Health publication number 80-23) as approved by the University of North Dakota animal care and use committee. Male Sprague-Dawley rats (200 to 250 g; Charles River Laboratories, Portage, MI, USA) were allowed to acclimate in our facility for at least two weeks prior to inclusion in the study and were maintained on a constant 12-hour light cycle and fed a standard laboratory chow (Purina 2018 Teklad Global) *ad libitum*. Rats were starved for at least 12 hours before surgeries and the first treatment with either GTA or water to normalize circulating levels of glucose and fatty acids [27].

In order to induce neuroinflammation, animals had cannulas (Model 3280PM, Plastics One, Roanoke, VA, USA) connected to subcutaneous osmotic mini-pumps (Model 2004, Durect Corporation, Cupertino, CA, USA) surgically implanted into the fourth ventricle of the brain as previously described [17]. The concentration of endotoxin used in these studies (5.0 ng/hour) was based on data showing that this concentration results in significant neuroglial activation and cholinergic cell loss above control rats [17], and is consistent with previous studies demonstrating a selective increase in arachidonic acid metabolism using this model [18,19]. During the infusion period, rats were treated daily with either GTA or water at a dose of 6 g/kg by gastric gavage using feeding tubes (Instech Solomon, Plymouth, PA, USA). The rats used for histone acetylation analysis were divided into four different groups: group one (*n *= 8) received an artificial cerebral spinal fluid (aCSF) infusion and were treated daily with water for 28 days (aCSF + H_2_O); group two (n = 7) received an aCSF infusion and daily treatment with GTA for 28 days (aCSF + GTA); group three (n = 8) received an LPS infusion and daily treatment with water for 28 days (LPS + H_2_O); and group four (n = 5) received an LPS infusion and daily treatment with GTA for 28 days (LPS + GTA). The rats used for IL-1β analysis were divided into three different treatment groups: group one (n = 6) received an aCSF infusion (aCSF), group two (n = 12) received an LPS infusion (LPS), and group three (n = 6) received an LPS infusion and daily treatment with GTA for 28 days (LPS + GTA). On the 28^th ^day of treatment, animals were anesthetized with isoflurane (Butler Animal Health Supply, Dublin, OH, USA) in an induction chamber for 1 minute and then killed by decapitation. Brains were immediately removed and flash-frozen by immersing in liquid nitrogen. The postmortem intervals for the brain did not exceed 1 minute. All samples were stored at -80°C until used.

### Tissue extraction

The nuclei isolation and acid extraction of histones were performed as described previously [23]. Rat brains used to measure IL-1β levels were homogenized using an ultrasonic dismembrator (Fisher Scientific, Waltham, MA, USA) in ice-cold 50 mM tris(hydroxymethyl)aminomethane (Tris; pH 7.4) buffer containing 150 mM sodium chloride, 1 mM ethylene glycol tetraacetic acid, 1 mM sodium orthovanadate, 5 mM zinc chloride, 10 mM sodium fluoride, 1 mM phenylmethylsulfonyl fluoride, Complete, ethylenediaminetetraacetic acid-free protease inhibitor cocktail (Roche Applied Science, Indianapolis, IN, USA), and 0.1% Igepal CA-630 as previously described [28]. The cytosolic portion of the samples was isolated by centrifugation at 4°C for 20 minutes (4,500 × g) [29].The cytosolic fractions were stored at -80°C until use. Protein concentration was measured using the Bradford method with BSA as standard [30].

### Western blot analysis

Equal amounts of protein; 5 μg for brain H3, 3.5 μg for brain H4, 50 μg for brain IL-1β and 4 μg for all brain HDAC were prepared by boiling samples in loading buffer composed of 95% Laemmli sample buffer and 5% 2-mercaptoethanol (Sigma). The separation of histones was performed using a 10% to 20% Tris-hydrochloride (Tris HCl) gel, the different HDACs on a 15% Tris-HCl gel and IL-1β on a 15% Tris-HCl gel with an electrophoresis separation of 100 volts for 2 hours. The electrophoretic transfer of proteins onto a 0.45-μm nitrocellulose membrane was performed at 100 volts for 90 minutes on ice. Primary antibodies were prepared in Tris-buffered saline containing Tween (TTBS; 20 mM Tris buffer, pH 7.4, 150 mM sodium chloride, 0.05% Tween 20) and 5% non-fat dried milk. The antibody concentrations used were: total H4, 1:1000; acetylated H4K5, 1:1000; acetylated H4K8, 1:1000; acetylated H4K12, 1:1000; acetylated H4K16, 1:1000; total H3, 1:500; acetylated H3K9, 1:800; acetylated H3K14, 1:1000; IL-1β, 1:5000; α-tubulin, 1:1500; and HDAC1, 2, 3, 4, 5 and 7, 1:750. All primary antibodies were incubated with the nitrocellulose membranes overnight at 4°C. The blots probed for all histones and HDAC5 and HDAC7 were conjugated with a HRP-linked goat anti-rabbit secondary antibody, and their dilutions were 1:3000 in TTBS for all histone blots and 1:1000 in TTBS for HDAC5 and HDAC7 blots. The blots probed with primary HDAC1, 2, 3 and 4 antibodies were conjugated with a horse anti-mouse, HRP-linked secondary antibody at a dilution of 1:1000. For the detection of IL-1β, the HRP-linked secondary antibody dilution was set at 1:20,000. The blots probed with α-tubulin antibody were conjugated with a HRP-linked goat anti-mouse immunoglobulin M secondary antibody at a dilution of 1:2000 in TTBS. Bands were visualized with a SuperSignal^® ^West Pico or West Femto Chemiluminescent Substrate (Pierce, Rockford, IL, USA) using a UVP Bioimaging System (Upland, CA, USA). Image capturing and analysis was performed with LabWorks™ imaging software (version 4.5; UVP). Western blot data of acetylated histones is expressed as of the ratio of the optical density of acetylated histone residues to the optical density of total histone. Western blot data of HDAC is expressed as the ratio of the optical density of the respective HDAC to the optical density of the loading control α-tubulin. Western blot data of IL-1β is expressed as the ratio of the optical density of IL-1β to the optical density of the loading control Ponceau S.[31]

### Quantitative real-time polymerase chain reaction

Brain cortex samples (50 to 100 mg each) were homogenized in 1 mL TRIzol^® ^reagent, using a Polytron homogenizer. Homogenized samples were incubated at room temperature for 5 minutes to permit the dissociation of nucleoprotein complexes before adding 0.2 mL of chloroform, shaking the tubes vigorously by hand, and incubating again at room temperature for 3 minutes. The samples were then centrifuged at 12,000 × g for 10 minutes at 4°C. After centrifugation, the upper clear aqueous phase containing RNA was transferred to fresh tubes, where the RNA was precipitated from the aqueous phase by mixing with 0.5 mL of isopropyl alcohol. The mix was incubated at room temperature for 10 minutes then centrifuged at 12,000 × g for 10 minutes at 4°C. The RNA pellet was washed once with 1 mL of 75% ethanol and centrifuged at 7,500 × g for 5 minutes at 4°C. At the end of the RNA extraction, the ethanol was decanted and the RNA pellet was allowed to air-dry at room temperature for 5 minutes before re-dissolving in 200 μL of nuclease-free water. One microgram of RNA per sample was used for cDNA synthesis using the iScript cDNA synthesis kit according to the manufacturer's instructions. Amplification was performed using 500 ng cDNA, 500 nM of each of the reverse and forward IL-1β and β-actin primers, and FastStart Universal SYBR Green Master in a final reaction volume of 50 μL, using a two-step cycling program of one cycle of 95°C for 10 minutes followed by 40 repeats of 95°C for 15 seconds and 60°C for 60 seconds in iCycler iQ Multicolor Real-Time PCR Detection System (Bio-Rad). The expression of IL-1β transcript amplified was normalized to the expression of β-actin. The amplification product was mixed with DNA Gel loading buffer 10 × (5 Prime, Gaithersburg, MD, USA), and run on 1% agarose gel at 100 volts for 1 hour. A TrackIt™ DNA ladder (Invitrogen, Grand Island, NY, USA) was used. PCR quantification was performed using the Livak formula [32].

### Histone deacetylases and histone acetyltransferases and activity assays

HDAC and HAT activities in the brain were measured using the colorimetric HDAC activity assay kit (Millipore, Billerica, MA, USA) and HAT activity assay kit (Abcam), respectively, according to the manufacturers' instructions and as described previously [23]. The colorimetric HDAC assay measures the total HDAC activity in a two-step procedure performed in a 96-well plate. In the first step, samples were incubated with the HDAC assay substrate, allowing deacetylation of the substrate. Next, the addition of an activator solution released p-nitroanilide from the deacetylated substrate or standard, monitored by spectrophotometric analysis. The colorimetric HAT activity assay depends on the acetylation of a peptide substrate by the active HAT; a process associated with the release of the free form of CoA. CoA serves as an essential coenzyme for the production of reduced nicotinamide adenine dinucleotide (NADH), which is measured using spectrophotometric analysis upon its reaction with a soluble tetrazolium dye. HDAC activity is expressed as the ratio of absorbance at 405 nm after 75 minutes of incubation at 37°C, normalized to the amount of nuclear extract used. HAT activity is expressed as the ratio of the absorbance at 450 nm after 4 hours of incubation at 37°C, normalized to the amount of nuclear extract used.

### Statistical analysis

A parametric one-way analysis of variance (ANOVA) followed by Tukey's post-hoc test or nonparametric one-way ANOVA followed by Dunn's post-hoc test were used as appropriately indicated to calculate statistical differences using GraphPad InStat statistical software (Version 3.06 for Windows, San Diego, CA, USA). All results are expressed as means ± SD with the significance threshold set at *P *≤ 0.05.

## Results

### Acetate supplementation and brain histone H3 and histone H4 acetylation

**Figure 1:**
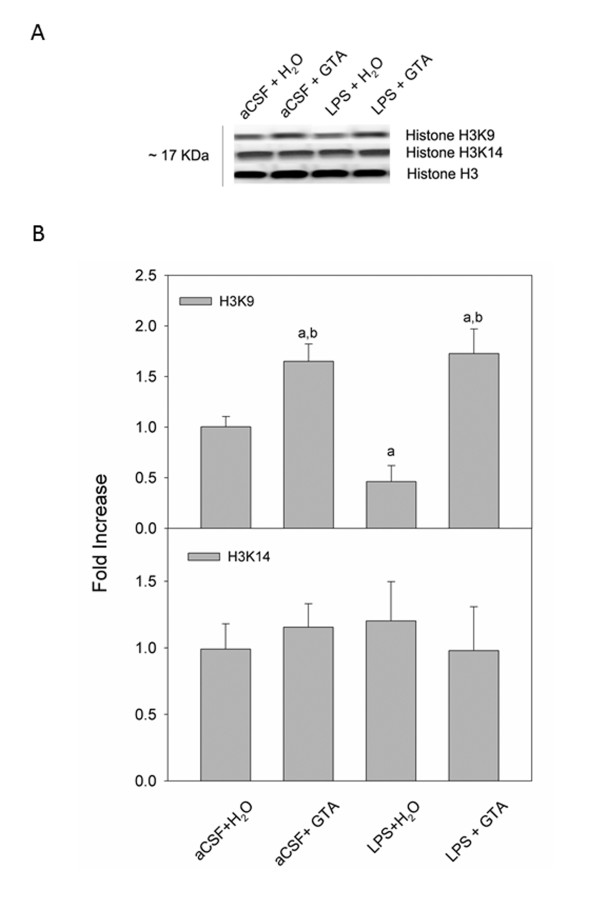
**Changes in the acetylation state of brain H3K9, and H3K14 in control rats (aCSF), rats subjected to neuroinflammation (LPS) and rats treated with either water (H_2_O) or glyceryl triacetate (GTA)**. **(A) **Representative images of the western blots. **(B) **Averaged proportion of brain H3K9 and H3K14, normalized to total H3. The data represent the means ± SD where statistical significance (a = compared to aCSF + H_2_O, and b = compared to LPS + H_2_O) was set at *P *≤ 0.05, as determined by one-way ANOVA followed by Tukey's post-hoc test.

The proportions of acetylated histone H3 and H4 were measured using Western blot analysis. Total H3, acetylated H3K9 and acetylated H3K14 were detected as protein bands corresponding to a molecular weight of 17 kDa (Figure 1A). Optical density analysis of acetylated H3 showed a 1.6-fold increase in the proportion of H3K9 in the two groups of rats subjected to acetate supplementation (aCSF + GTA) and (LPS + GTA), compared to controls (aCSF + H_2_O) (Figure 1B). Interestingly, the proportion of acetylated H3K9 was decreased by 2-fold in rats subjected to neuroinflammation and treated with water (LPS + H_2_O) and 3-fold when compared to rats that received acetate (aCSF + GTA and LPS + GTA). Neither acetate supplementation nor neuroinflammation altered the acetylation state of brain H3K14.

**Figure 2:**
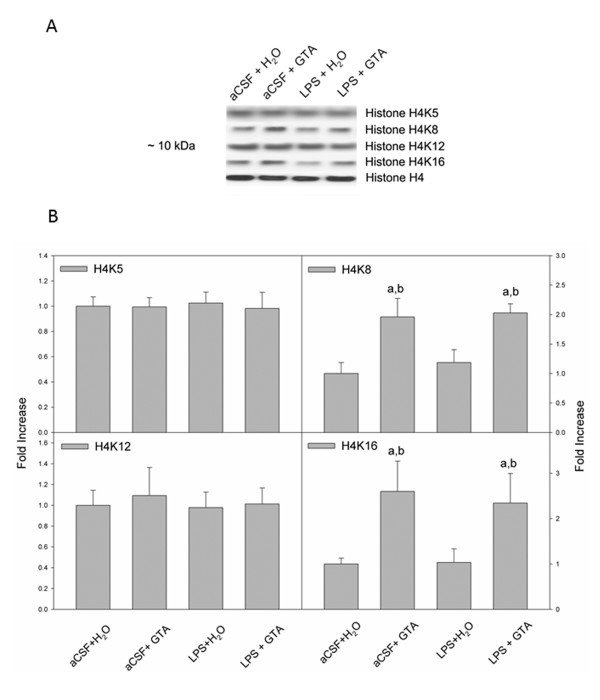
**Changes in the acetylation state of brain H4K5, H4K8, H4K12 and H4K16 in control rats (aCSF), rats subjected to neuroinflammation (LPS) and rats treated with either water (H_2_O) or glyceryl triacetate (GTA)**. **(A) **Representative images of the western blots **(B) **Averaged proportion of brain H4K5, H4K8, H4K12 andH4K16, normalized to total H4. The data represent the means ± SD where statistical significance (a = compared to aCSF + H_2_O, and b = compared to LPS + H_2_O) was set at *P *≤ 0.05, as determined by one-way ANOVA followed by Tukey's post-hoc test.

Total H4 and acetylated H4K5, H4K8, H4K12 and H4K16 were detected as a protein bands at 10 kDa which correspond to the molecular weight of these histones (Figure 2A). Similar to the proportional increases in acetylated H3K9, the proportions of acetylated H4K8 and H4K16 were significantly increased by 2-fold in rats subjected to acetate supplementation (aCSF + GTA and LPS + GTA; Figure 2B). However, unlike the proportion of acetylated H3K9, the proportion of acetylated H4K8 and H4K16 were not decreased in the rats subjected to neuroinflammation and treated with water (LPS + H_2_O) when compared to controls (aCSF + H_2_O). Neither acetate supplementation nor neuroinflammation altered the acetylation state of H4K5 or H4K12. These results suggest that acetate supplementation can selectively and positively regulate the acetylation of both histone H3 and H4 at specific acetylation sites. However, LPS-induced neuroinflammation only decreased the acetylation state of H3K9, which was effectively reversed with acetate supplementation.

### Acetate supplementation and brain histone deacetylase and histone acetyltransferase activities

**Figure 3:**
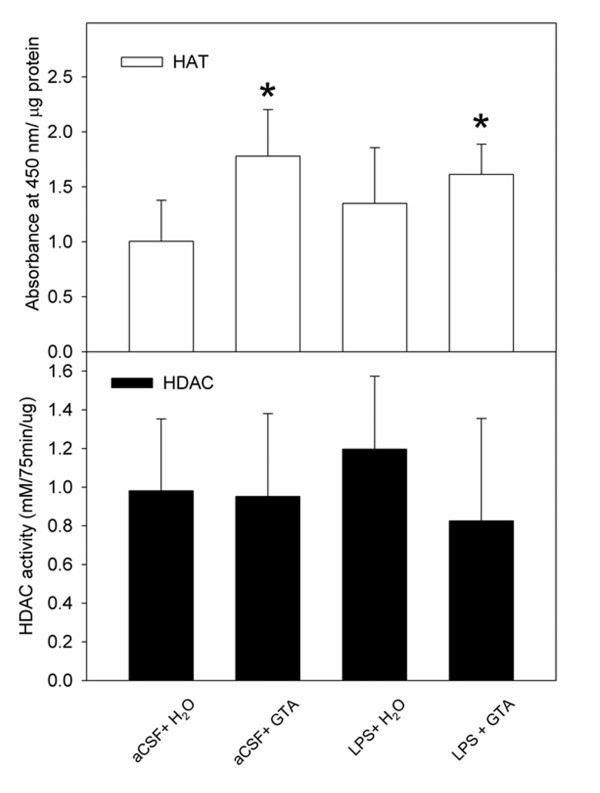
**The effect of long-term acetate supplementation on brain histone deacetylase and histone acetyltransferase activities in control rats (aCSF), rats subjected to neuroinflammation (LPS) and rats treated with either water (H_2_O) or glyceryl triacetate (GTA)**. **(A) **Represents changes in brain HAT, and **(B) **represents changes in HDAC activities. HAT enzyme activity is expressed as the means ± SD of absorbance reading at 450 nm after 4 hours of incubation at 37°C, normalized to the protein assayed (μg). HDAC enzyme activity is expressed as the means ± SD of absorbance at 405 nm after 75 minutes of incubation at 37°C, normalized to the protein assayed (μg). Statistical significance (*compared to aCSF + H_2_O controls) was set at *P *≤ 0.05, as determined by nonparametric one-way ANOVA followed by Dunn's post-hoc test.

Because the brain histone acetylation state is controlled by the activities of HAT and HDAC, we examined the effect that acetate supplementation had on brain HAT and HDAC activities in different test groups. Using commercially available HDAC and HAT assay kits, we found that acetate supplementation significantly increased, by 1.8- and 1.6-fold, the activity of brain HAT in the aCSF + GTA and LPS + GTA groups, respectively, compared to control aCSF + H_2_O rats (Figure 3). No changes in HAT activity were observed in rats subjected to neuroinflammation and treated with water (LPS + H_2_O). The HAT activity in the control sample was 35 ± 12 AU at 450 nm/ng protein, while the HAT activities in the aCSF + GTA and the LPS + GTA groups were 62 ± 14 and 56 ± 10 AU at 450 nm/ng protein, respectively. In parallel experiments, we did not find any changes in brain HDAC activity in any of the groups tested. These results suggest that long-term acetate supplementation, and not LPS-induced neuroinflammation, positively regulates brain HAT activity with no apparent effect on brain HDAC activity.

### Acetate supplementation and brain histone deacetylase expression

**Figure 4:**
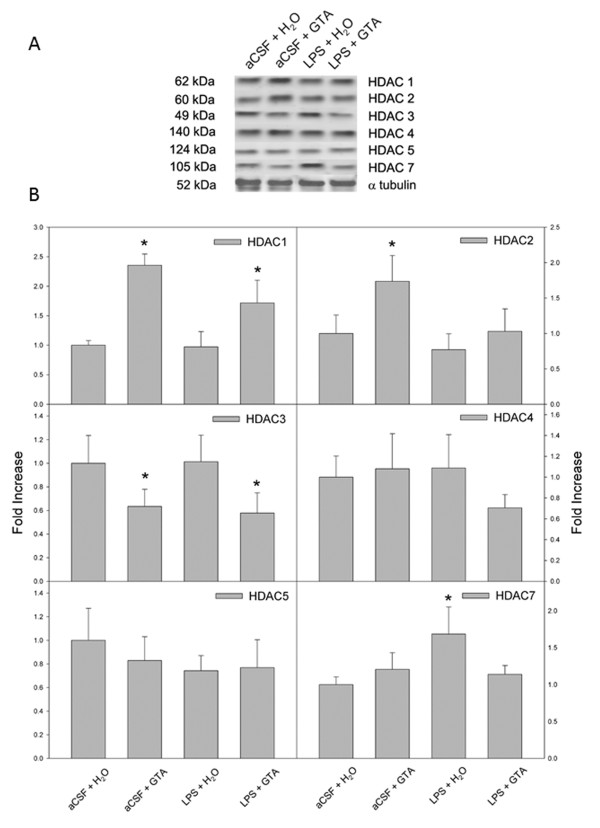
**The effect of long-term acetate supplementation on the expression of individual brain histone deacetylase in control rats (aCSF), rats subjected to neuroinflammation (LPS) and rats treated with either water (H_2_O) or glyceryl triacetate (GTA)**. **(A) **Representative images of the western blots. **(B) **Quantifications of HDAC1, 2, 3, 4, 5 and 7. Data in B represent means ± SD of the optical densities normalized to α-tubulin. Statistical significance (* compared to aCSF + H_2_O controls) was set at *P *≤ 0.05, as determined by one-way ANOVA followed by Tukey's post-hoc test for HDAC2, 3, 4 and 5, and nonparametric one-way ANOVA followed by Dunn's post-hoc test for HDAC1 and 7.

In an earlier study we found that a single oral dose of GTA decreases HDAC activity and reduces the expression of HDAC2 [23]. In the current study, however, we did not observe changes in HDAC activity, suggesting that the effects of long-term treatment differ substantially from a single oral dose. In order to further understand the effect that long-term acetate supplementation has on HDAC expression, we measured changes in the individual HDAC levels using western blot analysis. Optical density analysis showed that HDAC1 expression was significantly elevated by 2.4- and 1.7-fold in rats treated with GTA (aCSF + GTA and LPS + GTA; Figure 4B). A similar effect was found in HDAC2 expression where the level was significantly increased by 1.7-fold in rats treated with acetate (aCSF + GTA), but not in rats subjected to neuroinflammation (LPS + GTA). The expression levels of HDAC1 and HDAC2 were not altered by LPS-induced neuroinflammation treated with water (LPS + H_2_O). On the contrary, the levels of HDAC3 were significantly decreased by 40% in rats treated with acetate, (aCSF + GTA and LPS + GTA) as compared to control rats (aCSF + H_2_O). The HDAC3 expression level was not altered in rats subjected to neuroinflammation treated with water (LPS + H_2_O) as compared to control rats (aCSF + H_2_O). Most interestingly, the expression of HDAC7 was increased by 1.7-fold in rats subjected to neuroinflammation and treated with water (LPS + H_2_O), which was effectively reversed with acetate supplementation (aCSF + GTA and LPS + GTA). No changes were found in the expression levels of HDAC4 and HDAC5 in any of the groups tested. These results suggest the HDAC7 may play a positive role in the inflammatory process evoked by long-term LPS infusion that does respond to long-term acetate supplementation.

### Acetate supplementation and brain interleukin-1β expression

**Figure 5:**
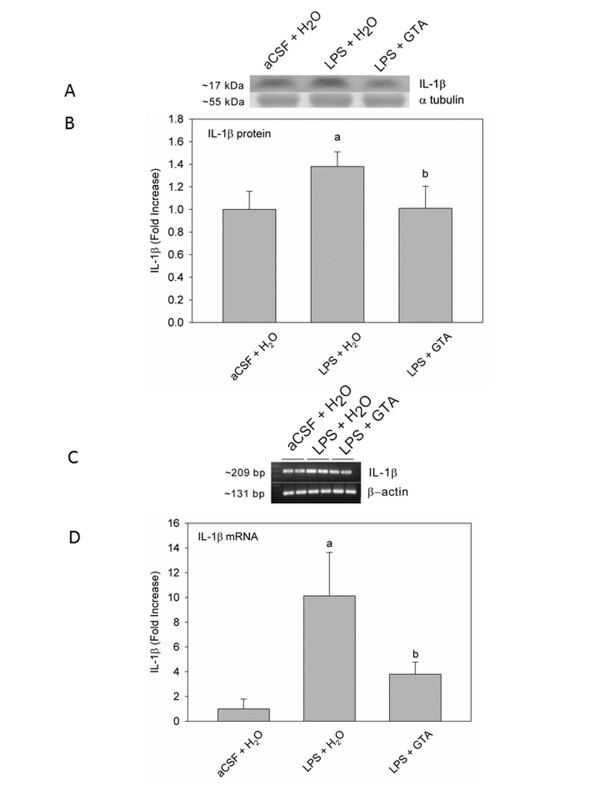
**The effect of long-term acetate supplementation on the expression of brain IL-1β in control rats (aCSF), rats subjected to neuroinflammation (LPS) and rats treated with either water (H_2_O) or glyceryl triacetate (GTA) as determined by Western blot analysis and quantitative real-time PCR**. **(A) **Western blot representative images of bands for IL-1β and α-tubulin and **(B) **shows the means ± SD of the normalized optical density of IL-1β protein. **(C) **Representative images of the bands for IL-1β and β-actin cDNA and **(D) **shows the means ± SD of the normalized amplified IL-1β cDNA. Statistical significance (a = compared to aCSF + H_2_O, and b = compared to LPS + H_2_O) was set at *P *≤ 0.05 (n = 6, per group), as determined by a one-way ANOVA followed by Tukey's post-hoc test.

To test whether acetate supplementation can reduce the expression of pro-inflammatory cytokines, and to provide insight into the anti-inflammatory mechanism by which acetate supplementation is effective in this model of neuroinflammation, we measured the effect that acetate treatment has on the protein and mRNA levels of brain pro-inflammatory cytokine IL-1β. In these experiments, we found protein bands corresponding to a molecular weight of 17 kDa corresponding to IL-1β (Figure 5A), and cDNA bands corresponding to 209 and 131 base pairs which correspond to the expected base pair size of IL-1β and β-actin, respectively (Figure 5C). In rats subjected to neuroinflammation (LPS + H_2_O), IL-1β protein and mRNA levels were significantly elevated by 1.3- and 10-fold, respectively, (Figure 5B,D). Further, the long-term acetate supplementation significantly reduced the expression level of IL-1β below that found in rats subjected to neuroinflammation (LPS), and equal to the control levels (aCSF).

## Discussion

Understanding the functional consequences of alterations in histone acetylation in the post-mitotic brain is important given the impact it can have on injury progression and resolution. We have found that long-term dietary acetate supplementation decreases neuroglia activation and cholinergic cell loss in a rat model of neuroinflammation [17] and that a single dose increases histone acetylation, decreases HDAC activity and decreases HDAC2 expression [23]. Because histone hyperacetylation is anti-inflammatory [24] and can alter gene expression [3], we propose that dietary acetate is anti-inflammatory by reducing pro-inflammatory gene expression. To begin to test this hypothesis we quantified the ability of long-term acetate supplementation to increase brain histone acetylation, to alter HAT and HDAC activities, and measured its ability to decrease the expression of the pro-inflammatory cytokine IL-1β. Unlike a single dose of GTA, long-term acetate supplementation increased HAT activity and had no effect on total brain HDAC activity, with variable effects on brain HDAC class I and II expression. In agreement with our hypothesis, neuroinflammation reduced the proportion of brain H3K9 acetylation by 50% and increased IL-1β protein and mRNA levels by 1.3- and 10- fold, respectively, all of which were effectively reversed with long-term acetate supplementation.

The preferential hyperacetylation of brain H4K16 is recognized as a central switch in higher-order chromatin structure [4]. When arranged into nucleosomal arrays, acetylated H4K16 inhibits the formation of higher-order 30-nm chromatin fibers and activates gene expression. The acetylation state at H4K16 is also an epigenetic hallmark for certain cancers, including leukemia, lymphoma and colorectal adenocarcinoma cell lines, in which H4K16 hyperacetylation is lost [33,34]. Moreover, H4K16 acetylation is thought to play a unique role in DNA repair as it is defective in aging and neurodegenerative disorders [5]. In this regard, both H4K16 and H4K8 acetylation were increased after acetate supplementation. These results suggest that acetate supplementation may be effective at attenuating neuroinflammation by altering higher-order chromatin structure and reducing pro-inflammatory gene expression. In support of this notion, H4K12 acetylation, which was not altered in our model, is implicated in gene silencing [35]. Therefore, these data suggest that LPS-induced neuroinflammation and acetate treatment do not silence gene expression, but rather shift gene expression to either a more pro- or anti-inflammatory state, respectively.

The effect of neuroinflammation on brain histone acetylation in parallel with the drug effect found in the control and treated rats suggest that H3K9 acetylation and possibly HDAC7 activity may be directly involved in injury progression in this model. For example, as outlined in Figure 1, LPS-induced neuroinflammation resulted in a direct reduction in H3K9 acetylation that was reversed with acetate supplementation. This finding is corroborated by the work of Zhang *et al. *showing that the use of an HDAC inhibitor 4-dimethylamino-N-[5-(2-mercaptoacetylamino) pentyl] benzamide (DMA-PB) decreases microglial activation in a rat model of traumatic brain injury, and that this anti-inflammatory effect is associated with increased histone H3 acetylation [36]. Further, the proportion of acetylated H3K9 is significantly lower in livers of aged rats, suggesting that the acetylation of H3K9 may be required to maintain essential cellular functions [37]. Similarly, we found that the expression of HDAC7 was increased in rats subjected to LPS-induced neuroinflammation, and was again reversed with long-term acetate supplementation. This suggests that mechanisms are in place that may account for injury- and treatment-specific changes in histone acetylation. In contrast, the acetylation state of H3K14 was not altered by acetate supplementation or LPS-induced neuroinflammation, suggesting that H3K14 acetylation was not related to treatment or neuroinflammation.

Another discrepancy between the effects of a single oral dose of acetate versus long-term acetate supplementation is that a single treatment decreases brain HDAC activity, whereas long-term supplementation had no effect on total brain HDAC activity, despite multiple alterations in the expression of individual HDAC. This can be explained by the fact that HDAC assays performed in this study measured overall HDAC activity and were reflective of the sum of all the individual HDAC. Given the differences in HDAC expression (that is, increased brain HDAC1, 2 and 7 and decreased brain HDAC3), we believe that the histone acetylation pattern found in this study is a reflection of the substrate specificity of the specific HDAC expressed after treatment, despite no overall change in total brain HDAC activity. We speculate that the increased expression of HDAC1 and HDAC2 is a physiological response to long-term acetate supplementation and an increase in brain acetyl-CoA levels. Further, HDAC activity does not depend solely on the expression HDAC, but rather works in concert with co-regulatory repressive complexes to modify catalytic activity [7]. For example, cloned HDAC and purified HDAC do not have deacetylating activity *in vitro *due to the lack of other protein complexes found *in vivo *[38]. Therefore, HDAC activity is not expected to increase unless the increase in HDAC expression is paralleled by an increase in the expression of co-regulatory complexes. Moreover, we do not exclude the possibility that other post-transcriptional or post-translational modifications of the expressed HDAC render them less active, resulting in no overall change in total brain HDAC activity.

To address non-specific effects of acetate on gene expression, we performed an RT-PCR array to determine the simultaneous changes in 84 genes involved in both the innate and adaptive immune response. In this study we found no significant changes (> 0.5-fold) in the expression of 81 genes measured. However, three genes did show more than a 2-fold change; adenosine receptor 2a, caspase 4 and IL-1 receptor antagonist (data not shown), suggesting that acetate supplementation can influence the expression of other genes involved in adaptive and innate immune responses. Clinical trials using GTA to induce acetate supplementation in patients with Canavan's disease show that it does not produce biochemical or metabolic abnormalities [39,40] and animal studies using larger doses of GTA are well-tolerated and cause no apparent toxicity [20].

IL-1β, among other cytokines, is expressed in the brain at low levels under physiological conditions and contributes to the control of metabolism [41], temperature regulation [42], synaptic plasticity and neuronal transmission [43]. However, IL-1β is produced in high levels in many pathological conditions that include ischemic stroke [44], Alzheimer's disease [45,46], Down syndrome [45], multiple sclerosis [47], Parkinson's disease [48], epilepsy [49], amyotrophic lateral sclerosis [50] and HIV-associated dementia [51]. This suggests that IL-1β has an important role in the progression of neuroinflammation [52]. Upon injury, activated microglia produce inflammatory mediators which lead to activation and proliferation of astrocytes. Likewise, activated astrocytes release inflammatory mediators, leading to further inter-glial communication, that if left unchecked results in neuronal bystander lysis [26]. IL-1β is a major signaling molecule and is involved in both neuronal-glial and inter-glial interactions that can increase microglial proliferation in mixed glial cultures, but not in isolated microglial cultures [53], bolstering the notion that microglial activation is at least in part dependent on interactions with neighboring astrocytes [54]. Therefore, disruption of the IL-1β system using anti-IL-1β antibodies, receptor blockade, or interfering with activation by enzymatic cleavage ameliorates neuroinflammation and delays neurodegeneration [55].

In this regard, in a mouse model of septic shock, the histone deacetylase inhibitor suberoylanilide hydroxamic acid (SAHA) increases H3K9 acetylation and inhibits TNF-α and IL-1β gene expression in lung tissue [56]. This supports the premise that the reduction in the proportion of H3K9 is indeed associated with the LPS-induced neuroinflammation, and that increased H3K9 acetylation can be linked to reducing the pro-inflammatory cytokine IL-1β expression. However, IL-1β expression in microglia is controlled directly by p38α mitogen-activated protein kinase (MAPK) downstream of the toll-like receptor 4 complex and is modulated by inhibitors selective for this kinase [57]. Further, p38α MAPK is associated with other regulatory kinases that are potentially modified post-transcription by acetylation reactions. For example, HDAC inhibition increases the acetylation of mitogen-activated protein kinase phosphatase-1 (MKP-1) that promotes complex formation between MKP-1 and p38α MAPK. This results in a reduction in phosphorylated p38α MAPK and a reduction in cytokine formation [58]. Mechanistically, it is not known whether the net anti-inflammatory effect of HDAC inhibition is the result of alterations in pro-inflammatory gene expression or a direct result of modulating the acetylation-state of accessory proteins involved in toll-like receptor signaling. In this regard, HDAC7 activity was increased in rats subjected to neuroinflammation and was effectively reversed by acetate supplementation. Therefore, it is not clear at this point as to whether the treatment effect found in this study on IL-1β expression is a direct result of decreasing p38α MAPK phosphorylation or an indirect effect by modulating gene expression.

Another metabolite that has anti-inflammatory and neuroprotective properties is pyruvate. The supplementation of pyruvate, or its aliphatic ester ethyl pyruvate, decreases LPS and hydrogen peroxide-induced microglial activation and promotes neuronal survival [59]. In addition, the administration of pyruvate provides protection against hippocampal neuronal injury after transient cerebral ischemia in rats [60]. Pyruvate also boosts extracellular brain glucose levels and decreases contusion volume and neuronal death in a rat model of traumatic brain injury [61]. Because acetate-derived acetyl-CoA can inhibit pyruvate dehydrogenase and lead to the accumulation of brain pyruvate, it is not unreasonable to suggest that the anti-inflammatory and neuroprotective effects of acetate may occur due to the accumulation of pyruvate. It will be interesting to test whether pyruvate, like acetate, can increase brain acetyl-CoA levels and alter histone acetylation and pro-inflammatory gene expression.

To examine whether acetate works through a mitochondrial process, we measured the effect of acetate on mitochondrial biogenesis but found no alteration in neuronal mitochondrial numbers after 28 days of acetate supplementation (unpublished data). This, however, does not exclude the possibility that acetate alters other mitochondrial processes such as the tricarboxylic acid cycle, the energy state or mitochondrial gene expression.

## Conclusion

In conclusion, long-term acetate supplementation reduced IL-1β expression by a mechanism that may involve a distinct site-specific pattern of histone acetylation and/or HDAC expression in the brain. Based on comparisons made between treatment groups, potentially key mechanistic targets of acetate supplementation include the LPS-induced changes in H3K9 acetylation, the expression of HDAC7 and the pro-inflammatory cytokine IL-1β. Thus physical epigenetic changes and/or direct changes in protein acetylation may help to explain the functional consequences of acetate supplementation found in this model of neuroinflammation.

## Abbreviations

aCSF: artificial cerebrospinal fluid; ANOVA: analysis of variance; AU: absorbance units; BSA: bovine serum albumin; CoA: coenzyme A; GTA: glyceryl triacetate; HAT: histone acetyltransferases; HDAC: histone deacetylases; HRP: horseradish peroxidase; H3K9: histone H3 acetylated at lysine 9; H3K14: histone H3 acetylated at lysine 14; H4K5: histone H4 acetylated at lysine 5; H4K8: histone H4 acetylated at lysine 8; H4K12; histone H4 acetylated at lysine 12; H4K16: histone H4 acetylated at lysine 16; IL: interleukin; kDa: kiloDaltons; LPS: lipopolysaccharide; MAPK: mitogen-activated protein kinase; MKP-1: mitogen-activated protein kinase phosphatase-1; PCR: polymerase chain reaction; RT: reverse transcriptase; TNF: tumor necrosis factor; Tris: tris(hydroxymethyl)aminomethane; TTBS: Tween containing tris buffer saline.

## Competing interests

The authors declare that they have no competing interests.

## Authors' contributions

MLS, MDS, HMH and TAR participated in the research design. The experiments and data analysis were performed by MLS, MDS and HMH. In addition, MLS and TAR wrote or contributed to the writing of the manuscript. All authors read and approved the final version of the manuscript.

## References

[B1] Cheung WL Briggs SD Allis CD Acetylation and chromosomal functions Curr Opin Cell Biol 2000 12 326 333 10.1016/S0955-0674(00)00096-X 10801466

[B2] Gorisch SM Wachsmuth M Toth KF Lichter P Rippe K Histone acetylation increases chromatin accessibility J Cell Sci 2005 118 5825 5834 10.1242/jcs.02689 16317046

[B3] Strahl BD Allis CD The language of covalent histone modifications Nature 2000 403 41 45 10.1038/47412 10638745

[B4] Shogren-Knaak M Ishii H Sun JM Pazin MJ Davie JR Peterson CL Histone H4-K16 acetylation controls chromatin structure and protein interactions Science 2006 311 844 847 10.1126/science.1124000 16469925

[B5] Li X Corsa CA Pan PW Wu L Ferguson D Yu X Min J Dou Y MOF and H4 K16 acetylation play important roles in DNA damage repair by modulating recruitment of DNA damage repair protein Mdc1 Mol Cell Biol 2010 30 5335 5347 10.1128/MCB.00350-10 20837706 PMC2976376

[B6] Hezroni H Sailaja BS Meshorer E Pluripotency-related, VPA-induced genome-wide H3K9 acetylation patterns in embryonic stem cells J Biol Chem 2011 286 35977 35988 10.1074/jbc.M111.266254 21849501 PMC3195619

[B7] de Ruijter AJ van Gennip AH Caron HN Kemp S van Kuilenburg AB Histone deacetylases (HDACs): characterization of the classical HDAC family Biochem J 2003 370 737 749 10.1042/BJ20021321 12429021 PMC1223209

[B8] Avalos JL Bever KM Wolberger C Mechanism of sirtuin inhibition by nicotinamide: altering the NAD(+) cosubstrate specificity of a Sir2 enzyme Mol Cell 2005 17 855 868 10.1016/j.molcel.2005.02.022 15780941

[B9] Marmorstein R Roth SY Histone acetyltransferases: function, structure, and catalysis Curr Opin Genet Dev 2001 11 155 161 10.1016/S0959-437X(00)00173-8 11250138

[B10] Kuo MH Brownell JE Sobel RE Ranalli TA Cook RG Edmondson DG Roth SY Allis CD Transcription-linked acetylation by Gcn5p of histones H3 and H4 at specific lysines Nature 1996 383 269 272 10.1038/383269a0 8805705

[B11] Howe L Auston D Grant P John S Cook RG Workman JL Pillus L Histone H3 specific acetyltransferases are essential for cell cycle progression Genes Dev 2001 15 3144 3154 10.1101/gad.931401 11731478 PMC312843

[B12] Aravalli RN Peterson PK Lokensgard JR Toll-like receptors in defense and damage of the central nervous system J Neuroimmune Pharmacol 2007 2 297 312 10.1007/s11481-007-9071-5 18040848

[B13] Lehnardt S Massillon L Follett P Jensen FE Ratan R Rosenberg PA Volpe JJ Vartanian T Activation of innate immunity in the CNS triggers neurodegeneration through a Toll-like receptor 4-dependent pathway Proc Natl Acad Sci USA 2003 100 8514 8519 10.1073/pnas.1432609100 12824464 PMC166260

[B14] Hauss-Wegrzyniak B Vraniak PD Wenk GL LPS-induced neuroinflammatory effects do not recover with time Neuroreport 2000 11 1759 1763 10.1097/00001756-200006050-00032 10852239

[B15] Hauss-Wegrzyniak B Dobrzanski P Stoehr JD Wenk GL Chronic neuroinflammation in rats reproduces components of the neurobiology of Alzheimer's disease Brain Res 1998 780 294 303 10.1016/S0006-8993(97)01215-8 9507169

[B16] Hauss-Wegrzyniak B Lukovic L Bigaud M Stoeckel ME Brain inflammatory response induced by intracerebroventricular infusion of lipopolysaccharide: an immunohistochemical study Brain Res 1998 794 211 224 10.1016/S0006-8993(98)00227-3 9622633

[B17] Reisenauer CJ Bhatt DP Mitteness DJ Slanczka ER Gienger HM Watt JA Rosenberger TA Acetate supplementation attenuates lipopolysaccharide-induced neuroinflammation J Neurochem 2011 117 264 274 10.1111/j.1471-4159.2011.07198.x 21272004 PMC3070819

[B18] Rosenberger TA Villacreses NE Hovda JT Bosetti F Weerasinghe G Wine RN Harry GJ Rapoport SI Rat brain arachidonic acid metabolism is increased by a 6-day intracerebral ventricular infusion of bacterial lipopolysaccharide J Neurochem 2004 88 1168 1178 10.1046/j.1471-4159.2003.02246.x 15009672

[B19] Lee H Villacreses NE Rapoport SI Rosenberger TA *In vivo *imaging detects a transient increase in brain arachidonic acid metabolism: a potential marker of neuroinflammation J Neurochem 2004 91 936 945 10.1111/j.1471-4159.2004.02786.x 15525347

[B20] Mathew R Arun P Madhavarao CN Moffett JR Namboodiri MA Progress toward acetate supplementation therapy for Canavan disease: glyceryl triacetate administration increases acetate, but not N-acetylaspartate, levels in brain J Pharmacol Exp Ther 2005 315 297 303 10.1124/jpet.105.087536 16002461

[B21] Arun P Madhavarao CN Moffett JR Hamilton K Grunberg NE Ariyannur PS Gahl WA Anikster Y Mog S Hallows WC Denu JM Namboodiri AM Metabolic acetate therapy improves phenotype in the tremor rat model of Canavan disease J Inherit Metab Dis 2010 33 195 210 10.1007/s10545-010-9100-z 20464498 PMC2877317

[B22] Arun P Ariyannur PS Moffett JR Xing G Hamilton K Grunberg NE Ives JA Namboodiri AM Metabolic acetate therapy for the treatment of traumatic brain injury J Neurotrauma 2010 27 293 298 10.1089/neu.2009.0994 19803785 PMC2824219

[B23] Soliman ML Rosenberger TA Acetate supplementation increases brain histone acetylation and inhibits histone deacetylase activity and expression Mol Cell Biochem 2011 352 173 180 10.1007/s11010-011-0751-3 21359531

[B24] Adcock IM HDAC inhibitors as anti-inflammatory agents Br J Pharmacol 2007 150 829 831 10.1038/sj.bjp.0707166 17325655 PMC2013887

[B25] Langley B Gensert JM Beal MF Ratan RR Remodeling chromatin and stress resistance in the central nervous system: histone deacetylase inhibitors as novel and broadly effective neuroprotective agents Curr Drug Targets CNS Neurol Disord 2005 4 41 50 10.2174/1568007053005091 15723612

[B26] Streit WJ Walter SA Pennell NA Reactive microgliosis Prog Neurobiol 1999 57 563 581 10.1016/S0301-0082(98)00069-0 10221782

[B27] Kargas G Rudy T Spennetta T Takayama K Querishi N Shrago E Separation and quantitation of long-chain free fatty acids in human serum by high-performance liquid chromatography J Chromatogr 1990 526 331 340 2361977 10.1016/s0378-4347(00)82517-7

[B28] Sohrabji F Peeples KW Marroquin OA Local and cortical effects of olfactory bulb lesions on trophic support and cholinergic function and their modulation by estrogen J Neurobiol 2000 45 61 74 10.1002/1097-4695(20001105)45:2<61::AID-NEU1>3.0.CO;2-L 11018768

[B29] Hulse RE Kunkler PE Fedynyshyn JP Kraig RP Optimization of multiplexed bead-based cytokine immunoassays for rat serum and brain tissue J Neurosci Methods 2004 136 87 98 10.1016/j.jneumeth.2003.12.023 15126049 PMC2801052

[B30] Bradford MM A rapid and sensitive method for the quantitation of microgram quantities of protein utilizing the principle of protein-dye binding Anal Biochem 1976 72 248 254 10.1016/0003-2697(76)90527-3 942051

[B31] Romero-Calvo I Ocon B Martinez-Moya P Suarez MD Zarzuelo A Martinez-Augustin O de Medina FS Reversible Ponceau staining as a loading control alternative to actin in western blots Anal Biochem 2010 401 318 320 10.1016/j.ab.2010.02.036 20206115

[B32] Livak KJ Schmittgen TD Analysis of relative gene expression data using real-time quantitative PCR and the 2(-Delta Delta C(T)) Method Methods 2001 25 402 408 10.1006/meth.2001.1262 11846609

[B33] Fraga MF Ballestar E Villar-Garea A Boix-Chornet M Espada J Schotta G Bonaldi T Haydon C Ropero S Petrie K Iyer NG Pérez-Rosado A Calvo E Lopez JA Cano A Calasanz MJ Colomer D Piris MA Ahn N Imhof A Caldas C Jenuwein T Esteller M Loss of acetylation at Lys16 and trimethylation at Lys20 of histone H4 is a common hallmark of human cancer Nat Genet 2005 37 391 400 10.1038/ng1531 15765097

[B34] Fraga MF Esteller M Towards the human cancer epigenome: a first draft of histone modifications Cell Cycle 2005 4 1377 1381 10.4161/cc.4.10.2113 16205112

[B35] Braunstein M Sobel RE Allis CD Turner BM Broach JR Efficient transcriptional silencing in *Saccharomyces cerevisiae *requires a heterochromatin histone acetylation pattern Mol Cell Biol 1996 16 4349 4356 8754835 10.1128/mcb.16.8.4349 PMC231433

[B36] Zhang B West EJ Van KC Gurkoff GG Zhou J Zhang XM Kozikowski AP Lyeth BG HDAC inhibitor increases histone H3 acetylation and reduces microglia inflammatory response following traumatic brain injury in rats Brain Res 2008 1226 181 191 18582446 10.1016/j.brainres.2008.05.085 PMC2652585

[B37] Kawakami K Nakamura A Ishigami A Goto S Takahashi R Age-related difference of site-specific histone modifications in rat liver Biogerontology 2009 10 415 421 10.1007/s10522-008-9176-0 18814051

[B38] Saha RN Pahan K HATs and HDACs in neurodegeneration: a tale of disconcerted acetylation homeostasis Cell Death Differ 2006 13 539 550 10.1038/sj.cdd.4401769 16167067 PMC1963416

[B39] Madhavarao CN Arun P Anikster Y Mog SR Staretz-Chacham O Moffett JR Grunberg NE Gahl WA Namboodiri AM Glyceryl triacetate for Canavan disease: a low-dose trial in infants and evaluation of a higher dose for toxicity in the tremor rat model J Inherit Metab Dis 2009 32 640 650 10.1007/s10545-009-1155-3 19685155

[B40] Segel R Anikster Y Zevin S Steinberg A Gahl WA Fisher D Staretz-Chacham O Zimran A Altarescu G A safety trial of high dose glyceryl triacetate for Canavan disease Mol Genet Metab 2011 103 203 206 10.1016/j.ymgme.2011.03.012 21474353

[B41] Grossberg AJ Zhu X Leinninger GM Levasseur PR Braun TP Myers MG Jr Marks DL Inflammation-induced lethargy is mediated by suppression of orexin neuron activity J Neurosci 2011 31 11376 11386 10.1523/JNEUROSCI.2311-11.2011 21813697 PMC3155688

[B42] Huang KF Huang WT Lin KC Lin MT Chang CP Interleukin-1 receptor antagonist inhibits the release of glutamate, hydroxyl radicals, and prostaglandin E(2) in the hypothalamus during pyrogen-induced fever in rabbits Eur J Pharmacol 2010 629 125 131 10.1016/j.ejphar.2009.11.060 19958757

[B43] Vitkovic L Bockaert J Jacque C "Inflammatory" cytokines: neuromodulators in normal brain? J Neurochem 2000 74 457 471 10646496 10.1046/j.1471-4159.2000.740457.x

[B44] Legos JJ Whitmore RG Erhardt JA Parsons AA Tuma RF Barone FC Quantitative changes in interleukin proteins following focal stroke in the rat Neurosci Lett 2000 282 189 192 10.1016/S0304-3940(00)00907-1 10717423

[B45] Griffin WS Stanley LC Ling C White L MacLeod V Perrot LJ White CL Araoz C Brain interleukin 1 and S-100 immunoreactivity are elevated in Down syndrome and Alzheimer disease Proc Natl Acad Sci USA 1989 86 7611 7615 10.1073/pnas.86.19.7611 2529544 PMC298116

[B46] Shaftel SS Griffin WS O'Banion MK The role of interleukin-1 in neuroinflammation and Alzheimer disease: an evolving perspective J Neuroinflammation 2008 5 7 10.1186/1742-2094-5-7 18302763 PMC2335091

[B47] McGuinness MC Powers JM Bias WB Schmeckpeper BJ Segal AH Gowda VC Wesselingh SL Berger J Griffin DE Smith KD Human leukocyte antigens and cytokine expression in cerebral inflammatory demyelinative lesions of X-linked adrenoleukodystrophy and multiple sclerosis J Neuroimmunol 1997 75 174 182 10.1016/S0165-5728(97)00020-9 9143252

[B48] Parish CL Finkelstein DI Tripanichkul W Satoskar AR Drago J Horne MK The role of interleukin-1, interleukin-6, and glia in inducing growth of neuronal terminal arbors in mice J Neurosci 2002 22 8034 8041 12223557 10.1523/JNEUROSCI.22-18-08034.2002 PMC6758077

[B49] Pernot F Heinrich C Barbier L Peinnequin A Carpentier P Dhote F Baille V Beaup C Depaulis A Dorandeu F Inflammatory changes during epileptogenesis and spontaneous seizures in a mouse model of mesiotemporal lobe epilepsy Epilepsia 2011 52 2315 2325 10.1111/j.1528-1167.2011.03273.x 21955106

[B50] Meissner F Molawi K Zychlinsky A Mutant superoxide dismutase 1-induced IL-1beta accelerates ALS pathogenesis Proc Natl Acad Sci USA 2010 107 13046 13050 10.1073/pnas.1002396107 20616033 PMC2919927

[B51] Zhao ML Kim MO Morgello S Lee SC Expression of inducible nitric oxide synthase, interleukin-1 and caspase-1 in HIV-1 encephalitis J Neuroimmunol 2001 115 182 191 10.1016/S0165-5728(00)00463-X 11282169

[B52] Basu A Krady JK Levison SW Interleukin-1: a master regulator of neuroinflammation J Neurosci Res 2004 78 151 156 10.1002/jnr.20266 15378607

[B53] Ganter S Northoff H Mannel D Gebicke-Harter PJ Growth control of cultured microglia J Neurosci Res 1992 33 218 230 10.1002/jnr.490330205 1333539

[B54] Kim JH Min KJ Seol W Jou I Joe EH Astrocytes in injury states rapidly produce anti-inflammatory factors and attenuate microglial inflammatory responses J Neurochem 2010 115 1161 1171 10.1111/j.1471-4159.2010.07004.x 21039520

[B55] Labow M Shuster D Zetterstrom M Nunes P Terry R Cullinan EB Bartfai T Solorzano C Moldawer LL Chizzonite R McIntyre KW Absence of IL-1 signaling and reduced inflammatory response in IL-1 type I receptor-deficient mice J Immunol 1997 159 2452 2461 9278338

[B56] Li Y Liu B Zhao H Sailhamer EA Fukudome EY Zhang X Kheirbek T Finkelstein RA Velmahos GC deMoya M Hales CA Alam HB Protective effect of suberoylanilide hydroxamic acid against LPS-induced septic shock in rodents Shock 2009 32 517 523 10.1097/SHK.0b013e3181a44c79 19295477

[B57] Bachstetter AD Xing B de Almeida L Dimayuga ER Watterson DM Van Eldik LJ Microglial p38alpha MAPK is a key regulator of proinflammatory cytokine up-regulation induced by toll-like receptor (TLR) ligands or beta-amyloid (Abeta) J Neuroinflammation 2011 8 79 10.1186/1742-2094-8-79 21733175 PMC3142505

[B58] Cao W Bao C Padalko E Lowenstein CJ Acetylation of mitogen-activated protein kinase phosphatase-1 inhibits Toll-like receptor signaling J Exp Med 2008 205 1491 1503 10.1084/jem.20071728 18504304 PMC2413043

[B59] Kim JB Yu YM Kim SW Lee JK Anti-inflammatory mechanism is involved in ethyl pyruvate-mediated efficacious neuroprotection in the postischemic brain Brain Res 2005 1060 188 192 10.1016/j.brainres.2005.08.029 16226231

[B60] Lee JY Kim YH Koh JY Protection by pyruvate against transient forebrain ischemia in rats J Neurosci 2001 21 RC171 11588201 10.1523/JNEUROSCI.21-20-j0002.2001 PMC6763857

[B61] Fukushima M Lee SM Moro N Hovda DA Sutton RL Metabolic and histologic effects of sodium pyruvate treatment in the rat after cortical contusion injury J Neurotrauma 2009 26 1095 1110 10.1089/neu.2008.0771 19594384 PMC2848946

